# When life gives you limes… it gives you Candida: a case of lime-contaminated IV injection

**DOI:** 10.1093/omcr/omag077

**Published:** 2026-05-24

**Authors:** Priscila Lopez, Thupten Lama, Parul Kodan, Vel Sivapalan

**Affiliations:** Department of Infectious Diseases, Harlem Hospital Centre, NY, NYC 10037, US; Department of Infectious Diseases, Harlem Hospital Centre, NY, NYC 10037, US; Department of Infectious Diseases, Harlem Hospital Centre, NY, NYC 10037, US; Department of Infectious Diseases, Harlem Hospital Centre, NY, NYC 10037, US

**Keywords:** candida, candidemia, intravenous drug use, fungal infection

## Abstract

We present a case of a 29-year-old male with a history of intravenous drug use, who developed candidemia after injecting crack cocaine mixed with lime juice. The patient initially presented with sepsis, and the preliminary blood cultures grew *Candida krusei*. Upon further investigation, the final blood culture report also grew *Candida lusitaniae*. He was started on Caspofungin and completed a 14-day antifungal course, with a favorable outcome. This case may represent a rare instance of polymicrobial nonalbicans candidemia linked to lime juice injection, unlike typical acidifier-associated infections, which usually involve a single organism. This case underscores the elevated risk of invasive polymicrobial fungal infections, particularly candidemia, in intravenous drug users who use lime juice as a solvent, highlighting the need for early diagnosis and prompt treatment.

## Introduction

In the mid-1980s, crack cocaine was developed for smoking. While it is insoluble in water and unsuitable for injection, individuals often use weak acids such as lemon juice, lime juice, vinegar, and ascorbic acid to enhance the solubility of crack cocaine, making it suitable for injection [1]. Different studies have concluded that contaminated juice is a source for candidemia in intravenous drug users [1–3].

This case highlights a rare instance of polymicrobial non-*albicans Candida* candidemia associated with lime juice injection, thereby extending previously described acidifier-associated fungal infections and emphasizing an underrecognized possible mechanism of invasive disease transmission.

Invasive fungal infections caused by candidiasis are significant contributors to morbidity and mortality in both adults and children [4]. *Candida albicans* is responsible for approximately 50% of candidemia cases [5]. A study of 2019 candidemia cases between 2004 and 2008 found *C. albicans* accounted for 46% of the infections, followed by *C. glabrata* (26%), *Candida parapsilosis* (16%), *C. tropicalis* (8%), and *Candida krusei* (3%) [6]. *Candida lusitaniae* has been reported to account for 19.3% of fungemia cases in immunocompromised patients and 1.7% of candidiasis cases in the general population [7].

Key risk factors for invasive *Candida* infections include immunosuppression, the presence of central venous catheters, use of broad-spectrum antibiotics, renal disease requiring dialysis, and genitourinary or gastrointestinal surgeries or procedures [4]. *C. krusei* and *lusitaniae* candidemia were most frequently linked to prior antifungal use, hematologic malignancies, stem cell transplantation, neutropenia, and corticosteroid therapy [5–9].

Early diagnosis and prompt, effective initial treatment are critical for improving infection outcomes [7, 8, 10]. Echinocandins are typically the preferred first-line treatment for most cases of invasive candidiasis. The recommended duration of systemic antifungal therapy for candidemia is a minimum of 14 days, following the clearance of *Candida* spp. from the blood and the resolution of all infection-related symptoms and signs [10].

This case underscores the importance of considering contaminated acidic diluents as a source of invasive fungal infection in people who inject drugs and highlights the need for early recognition and harm reduction strategies.

## Case presentation

A 29-year-old male with a medical history of substance use disorder, intravenous drug use, bipolar disorder, anemia, and a prior Hepatitis C infection presented two days after an emergency department visit for a mechanical fall, which resulted in lacerations to the chin, shoulders, and knees. At that time, he was febrile with a temperature of 101.8°F, tachycardic (heart rate 103 bpm), normotensive, and not in respiratory distress. A sepsis workup was initiated, and broad-spectrum antibiotics were administered. However, the patient left the hospital against medical advice before further evaluation could be completed.

He returned to the hospital with complaints of chest pain and shortness of breath, which had been ongoing for the past two days. He denied fever, chills, nausea, vomiting, headache, vision changes, palpitations, dizziness, lightheadedness, cough, back pain, changes in bowel movements, or weight loss. The patient reported injecting cocaine mixed with lime juice and confirmed that he does not share needles but reuses them.

**Figure 1:**
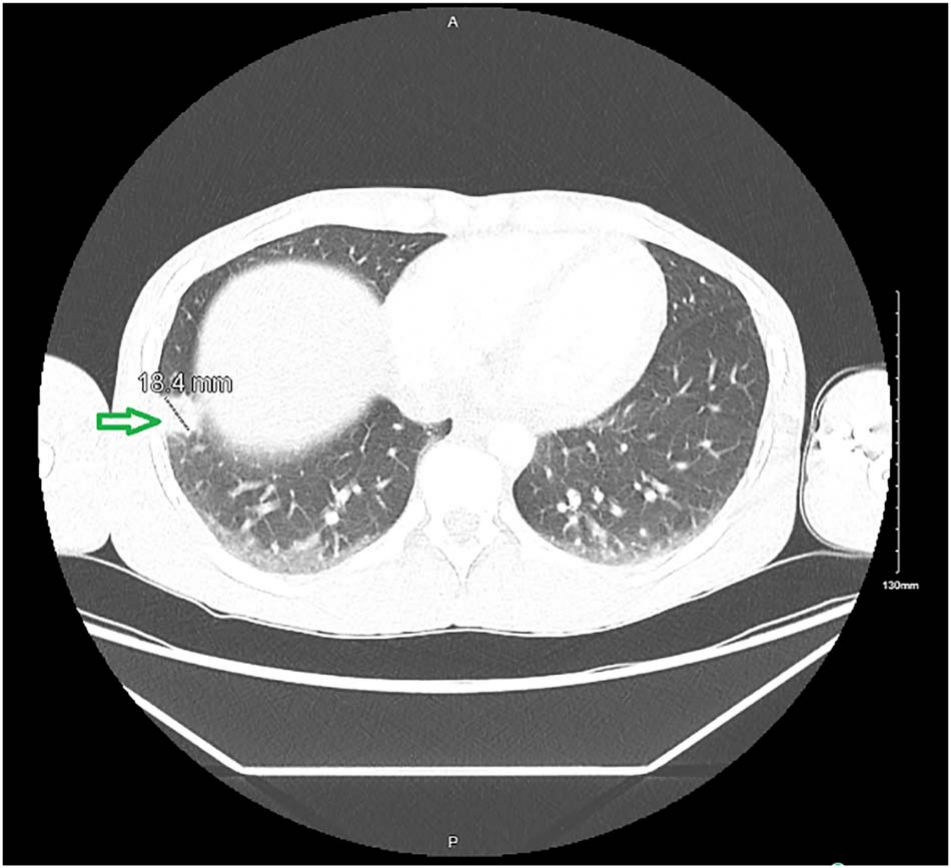
Axial CT scan of the chest demonstrating bilateral lung parenchyma. A focal peripheral lesion (green arrow) is seen in the left lung measuring approximately 18.4 mm, located subpleurally. Surrounding lung fields show patchy opacities, suggestive of associated inflammatory or infectious changes.

On presentation, the patient was hemodynamically stable (temperature 97.7°F, heart rate 71 bpm, respiratory rate 16 breaths/min, blood pressure 130/73 mmHg, and oxygen saturation 99%). Laboratory testing revealed thrombocytopenia, normocytic anemia, and a negative HIV screening (Table 1). Preliminary reports from blood cultures grew *C. krusei*, with susceptibilities pending. A chest X-ray was unremarkable, but a computed tomography (CT) scan showed an spiculated nodule in the right lower lobe (Fig. 1).

**Table 1:** Blood culture isolates and antifungal susceptibility profile.

Blood cultures: Growth of *Candida krusei* and *Candida lusitaniae* in aerobic and anaerobic bottles.				
Blood culture susceptibilities (*Minimum inhibitory concentrations (MICs) for antifungal agents are shown for each isolate)*				
Antifungal	*C. lusitaniae*		*C. krusei*	
	Yeast MIC	Interpretation	Yeast MIC	
Amphotericin B	1	No interpretation^a^	1	No interpretation^a^
Anidulafungin	0.12	No interpretation^b^	0.06	Sensitive^c^
Caspofungin	0.25	No interpretation^b^	0.25	Sensitive^c^
Fluconazole AF	2	No interpretation^d^	-	
Micafungin	0.12	No interpretation^b^	0.12	Sensitive^c^
Posaconazole	0.06	No interpretation^a^	0.5	No interpretation^a^
Voriconazole AF	0.03	No interpretation^b^	0.5	Sensitive^c^

^a^For Candida species, no interpretation is available for any in vitro susceptibility testing. Susceptibility interpretations are limited by the absence of established Clinical and Laboratory Standards Institute (CLSI) breakpoints for several antifungals and should be interpreted in the appropriate clinical context.

^b^For this antifungal agent, the data are based largely on experience with non-neutropenic patients with candidemia. The clinical relevance of this antifungal agent in other settings is uncertain.

^c^For this antifungal agent, the data are based largely on experience with non-neutropenic patients with candidemia. The clinical relevance of this antifungal agent in other settings is uncertain.

^d^Fluconazole: Susceptibility depends on achieving the maximum possible blood level. Doses higher than the standard dosing amount (6 mg/kg/day) may be needed in adults with normal renal function and body habitus.

Laboratory evaluation showed a white blood cell count of 4.8 × 10^3^/μl, hemoglobin of 12.2 g/dl, and mild thrombocytopenia with platelets of 110 × 10^3^/μl. Renal function was preserved with a BUN of 18 mg/dl and creatinine of 1.0 mg/dl. Infectious workup revealed reactive hepatitis C antibody with undetectable HCV RNA, while hepatitis B and HIV testing were negative. Fungal markers were notable for markedly elevated β-D-glucan (>500 pg/ml), with negative cryptococcal antigen and galactomannan. Additional testing, including Quantiferon, Histoplasma urine antigen, and sputum AFB, was negative.

The patient was started on broad-spectrum antibiotics and Caspofungin while awaiting final blood culture results susceptibilities. Despite this treatment, he left again against medical advice but was readmitted to another facility due to worsening chest tightness. There, Caspofungin was continued. Final results of blood cultures reported *C. krusei* and *C. lusitaniae* (Table 1). For the right lung nodule, the concern was septic emboli. Repeated chest CT demonstrated a decrease in size of the lung nodule to 0.8 cm.

Transthoracic and transesophageal echocardiography were negative for endocardial vegetations. The patient had no vision changes, nor eye related symptoms for which ophthalmological evaluation was deferred. Consideration was given to alternative endovascular sources, including injection site associated septic thrombophlebitis; however, patient had no definite signs or symptoms and targeted peripheral venous imaging was not undertaken, although the impact on management would likely have been minimal in the setting of ongoing clinical and radiographic improvement. The patient completed a 14-day course of antifungal therapy after clearing fungemia. He was counseled on substance use cessation and was discharged to a shelter with infectious disease and primary care follow-up, as well as a referral for substance dependency treatment.

## Discussion

This case highlights an emerging and underrecognized route of fungal bloodstream infection associated with the injection of crack cocaine dissolved in acidic solutions. The use of weak acids such as lemon or lime juice, vinegar, and ascorbic acid to solubilize crack cocaine is increasingly documented among people who inject drugs [11, 12]. These agents, although inexpensive and readily available, are non-sterile and often contaminated with environmental microorganisms [12, 13]. The practice not only facilitates injection but also causes endothelial damage, providing a direct portal for microbial invasion.

Several studies have reported severe complications related to this behavior, including localized abscesses, endophthalmitis, bacteremia, and candidemia [11–14]. Among these, fungal bloodstream infections represent one of the most serious outcomes. The current literature indicates that *C. albicans* remains the predominant species isolated in such cases; however, non-albicans species, including *C. parapsilosis*, *C. krusei*, and *C. lusitaniae*, are increasingly encountered, often displaying variable antifungal susceptibility profiles [4–7]. The occurrence of *C. krusei* and *C. lusitaniae* in our patient is particularly noteworthy given their inherent or acquired resistance to azoles, which complicates management and may contribute to higher morbidity and mortality [5, 7].

The mechanism of infection in such cases is likely multifactorial. We propose a potential hypothesis that acidic solutions cause chemical irritation and vascular injury, leading to endothelial disruption that facilitates the translocation of yeast into the bloodstream. Additionally, lime and lemon juice can serve as nutrient-rich media that support fungal growth, especially when stored at ambient temperatures [11, 12]. The repeated use of shared paraphernalia and non-sterile water sources further increases the risk of contamination. Even though *C. albicans* has been cultured from lime juice [12, 13], this case demonstrates that other Candida species may also be introduced through similar routes.

Epidemiologic data underscore the growing significance of invasive fungal infections in drug users. CDC surveillance reports that up to one-third of candidemia cases in some U.S. regions are associated with injection drug use [14]. This trend parallels the substance-use epidemic and reflects high-risk injection practices, limited access to sterile supplies, and delays in seeking care.

From a therapeutic standpoint, early identification of *Candida* species is essential, as *C. krusei* is intrinsically fluconazole-resistant, and *C. lusitaniae* may exhibit reduced susceptibility to amphotericin B [5–9]. The favorable response to caspofungin in this patient underscores the role of echinocandins in treating suspected non-albicans candidemia [10].

Beyond individual management, this case raises important public health considerations. The use of household acidic substances for drug preparation is a preventable risk factor. Harm-reduction programs should incorporate education on the dangers of acidifiers and promote access to sterile water or citric acid sachets [12, 13]. Integrating fungal infection prevention into broader substance-use care and strengthening laboratory capacity remain essential.

## Conclusion

In summary, this case expands the known spectrum of fungal infections associated with injection drug use, representing a rare instance of polymicrobial non-*albicans* candidemia associated with lime juice injection, underscores *Candida non-albicans* as a potential pathogen transmitted through contaminated acidic diluents, and highlights the importance of harm-reduction outreach.
